# Acute laryngotracheitis caused by COVID-19: A case report and literature review

**DOI:** 10.1016/j.ijscr.2022.107074

**Published:** 2022-04-12

**Authors:** Alhanouf A. Alhedaithy, Islam Salah Murad, Nada Aldabal

**Affiliations:** King Fahad Medical Military Complex, Dhahran, Saudi Arabia

**Keywords:** COVID-19, coronavirus disease 2019, SARS-CoV-2, severe acute respiratory syndrome coronavirus 2, HPIV, human parainfluenza virus, ICU, intensive care unit, ED, emergency department, Case report, Coronavirus disease 2019, Severe acute respiratory syndrome coronavirus 2, Laryngotracheitis, Otorhinolaryngology

## Abstract

**Introduction:**

Coronavirus disease 2019 (COVID-19) is an infectious disease caused by severe acute respiratory syndrome coronavirus 2 (SARS-CoV-2). Laryngotracheitis (croup) is a rare manifestation of COVID-19 in adults.

**Presentation of case:**

A 52-year-old female presented to the emergency department (ED) with shortness of breath and inspiratory stridor.

**Clinical findings and investigations:**

Physical examination of the head and neck revealed a congested posterior pharyngeal wall. Laryngeal endoscopy with a 70-degree rigid endoscope demonstrated an edematous, bilaterally moving vocal cords. Chest radiographs showed tapering of the upper trachea (the “steeple” sign), which is observed in parainfluenza-associated croup infections.

**Interventions and outcome:**

The patient was admitted to the intensive care unit (ICU) for close observation for possible airway compromise and the need for intubation. Upon which, she tested positive for COVID-19 by polymerase chain reaction testing of nasopharyngeal samples. A regimen of ceftriaxone, nebulized racemic epinephrine, and dexamethasone was initiated.

**Conclusion:**

During the current COVID-19 pandemic, early diagnostic testing for SARS-Cov-2 are strongly recommended even when symptoms are not typical of COVID-19.

## Highlights

• A 52-year-old woman presented to the ER with a two-day history of shortness of breath and inspiratory stridor.
• The patient was admitted to the ICU for possible surgical airway. she tested positive for COVID-19 by polymerase chain reaction testing of nasopharyngeal samples.
• A regimen of ceftriaxone, nebulized racemic epinephrine, and dexamethasone was initiated.
• The patient’s symptoms started to improve over the next few days. On day 4 of hospitalization, she moved to a level 1 bed, and a week later, she was discharged
• Early diagnostic testing for SARS-Cov-2 are strongly recommended even when symptoms are not typical of COVID-19

## 1 Introduction

This work has been reported in line with the SCARE criteria [1].

Coronavirus disease 2019 (COVID-19) is an infectious disease caused by severe acute respiratory syndrome coronavirus 2 (SARS-CoV-2). The disease was first identified in Wuhan, China, in December 2019 [2], and it spread rapidly throughout the world. The World Health Organization declared the outbreak a Public Health Emergency of International Concern on January 30, 2020, and a pandemic on March 11, 2020 [3].

As of January 25, 2022, more than 346 million cases of COVID-19 have been reported globally, of which more than 5.5 million have been fatal [4]. The clinical signs may appear 2–14 days after exposure (according to estimates of the incubation period of SARS-CoV-2). These signs include fever, sore throat, cough, and myalgia, and some patients have had symptoms of gastrointestinal infection [5].

“Laryngotracheitis” (croup) is a term for a heterogeneous group of illnesses that affect the larynx, the trachea, and the bronchi. Human parainfluenza viruses (HPIVs) account for almost 75% of all cases of croup. Signs and symptoms of HPIV infection are indistinguishable from those of other viral infections. HPIV infection may result in severe illness and deterioration of lung function, and prolonged hospitalization, intensive care unit (ICU) treatment, and mechanical ventilation may be necessary [7]. We describe the case of a patient in whom acute laryngotracheitis was a manifestation of COVID-19.

## 2 Presentation of case

The patient was a 52-year-old female nonsmoker with a history of hypertension (controlled with amlodipine, 10 mg once daily), type 2 diabetes mellitus (controlled with metformin, 500 mg thrice daily, and linagliptin, 5 mg once daily), dyslipidemia (controlled with atorvastatin, 10 mg once daily), and Sheehan syndrome (controlled with levothyroxine, 100 μg once daily, and hydrocortisone, 10 mg once daily). She presented to the emergency department at King Fahad Medical Military Complex in Al Dhahran, Saudi Arabia, with shortness of breath. The patient had been in her usual state of health until 2 days earlier, when she started experiencing exertional dyspnea with hoarseness and stridor. The patient was febrile upon presentation to the emergency department, with a temperature of 39.7 °C. She had received two doses of the COVID-19 vaccine, the second administered 5 months before presentation. She denied headache, cough, sore throat, hemoptysis, symptoms of gastrointestinal infection, and decrease in her level of consciousness. She also denied weight loss, night sweats, and pruritus, and she had no history of recent foreign travel or close contact with sick patients.

**Fig. 1:**
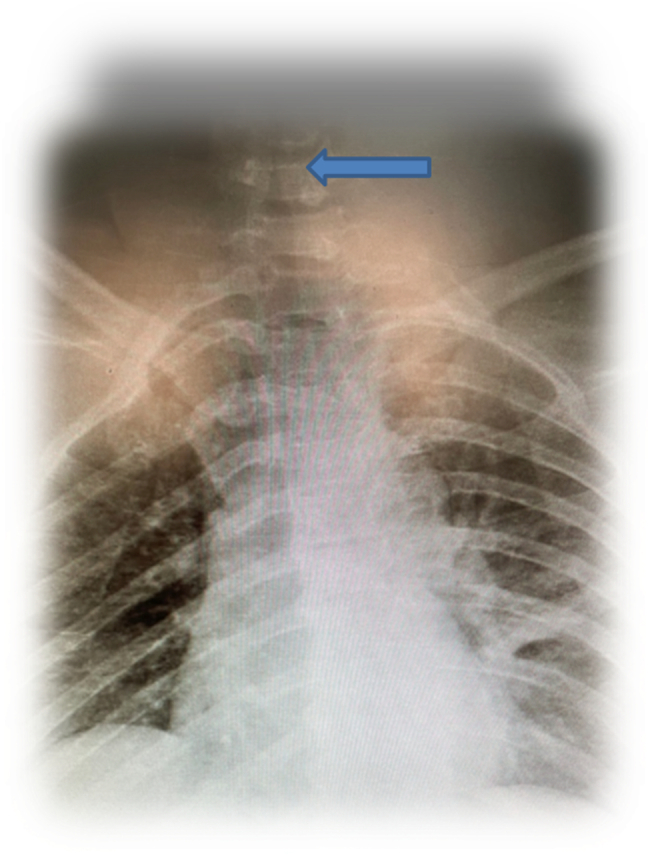
Steeple sign (arrow).

During physical examination, the patient exhibited inspiratory stridor; a nasal cannula was placed, and 2 L of oxygen was administered. Throat examination revealed congestion in the posterior pharyngeal wall. Laryngeal endoscopy with a 70-degree rigid endoscope revealed edematous, bilaterally moving vocal cords. Chest radiographs showed tapering of the upper trachea (the “steeple” sign), which is observed in parainfluenza-associated croup infections (Fig. 1). The complete blood cell count was normal, the C-reactive protein level was 108 mg/L, and her arterial blood gas measurements included a pH of 7.36, a base deficit of −4.6 mmol/L, and a partial pressure of oxygen of 54.2 mmHg. She was then transferred to the ICU for close observation for possible airway compromise and the need for intubation. Upon her admission to the ICU, she tested positive for COVID-19 by polymerase chain reaction testing of nasopharyngeal samples, which were negative for all other respiratory pathogens, including the most common causes of croup.

A regimen of ceftriaxone, nebulized racemic epinephrine, and dexamethasone was initiated. The patient's symptoms started to improve over the next few days. On day 4 of hospitalization, she moved to a level 1 bed, and a week later, she was discharged.

## 3 Discussion

Upper airway infections caused by viruses have various manifestations, ranging from dysphonia to fulminant airway compromise. Croup is usually associated with parainfluenza, rhinovirus, and respiratory syncytial virus infections. According to a literature review, less common viruses (herpes simplex, herpes zoster, and human immunodeficiency viruses) have been found to cause laryngotracheitis [8], [9]. Croup has been a presenting feature of COVID-19 in several reported cases in children [10], [11], [12], [13]. To our knowledge, however, laryngotracheitis has not previously been formally reported in adults with COVID-19.

The key finding in our patient was a presentation characteristic of HPIV-induced croup, hoarseness, respiratory distress, and stridor. Treatment included dexamethasone and nebulized racemic epinephrine. Pathogen testing is usually not indicated for croup because it is diagnosed through clinical assessment; however, “COVID-19 croup” testing should be considered because of its prognostic significance and the implications for prolonged quarantine.

Early consideration of COVID-19–related laryngotracheitis is important especially because airway complications may occur and because of the potential need for intubation and tracheostomy. In laryngotracheitis, the insertion of tracheostomy tubes may be problematic because of narrowing of the airway, thickening of the mucosa, and increase in local secretions [14].

## 4 Conclusion

During the current COVID-19 pandemic, after careful consideration of a patient's clinical condition, infection control measures and early diagnostic testing for SARS-Cov-2 are strongly recommended even when symptoms are not typical of COVID-19.

## Ethical approval

The case report was approved by the institutional review board at King Fahad Medical Military Complex, Dhahran, Saudi Arabia.

## Author contribution

Alhanouf Alhedaithy: Writing-original draft.

Islam Murad: Writing-review & editing.

Nada Aldabal: Writing-review & editing, Supervision

## Registration of research studies

None.

## Sources of funding

None.

## Consent

Written informed consent was obtained from the patient for publication of this case report and accompanying images. A copy of the written consent is available for review by the Editor-in-Chief of this journal on request.

## Guarantor

Alhanouf Alhedaithy.

## Provenance and peer review

Not commissioned, externally peer-reviewed.

## Declaration of competing interest

None.
